# Impact of Wide Local Excision on Melanoma Patient Survival: A Population-Based Study

**DOI:** 10.3389/fpubh.2022.806934

**Published:** 2022-03-31

**Authors:** Alessandra Buja, Massimo Rugge, Giovanni Damiani, Giuseppe De Luca, Manuel Zorzi, Riccardo Fusinato, Chiara De Toni, Antonella Vecchiato, Paolo Del Fiore, Francesca Falasco, Romina Spina, Carlo Riccardo Rossi, Simone Mocellin

**Affiliations:** ^1^Department of Cardiologic, Vascular and Thoracic Sciences, and Public Health, University of Padova, Padua, Italy; ^2^Veneto Tumor Registry, Azienda Zero, Padua, Italy; ^3^Pathology and Cytopathology Unit, Department of Medicine-DIMED, University of Padova, Padua, Italy; ^4^Clinical Dermatology, Istituto di Ricovero e Cura a Carattere Scientifico (IRCCS) Istituto Ortopedico Galeazzi, Milan, Italy; ^5^Department of Biomedical, Surgical and Dental Sciences, University of Milan, Milan, Italy; ^6^Department of Pharmaceutical and Pharmacological Sciences, Ph.D. Degree Program in Pharmacological Sciences, University of Padua, Padua, Italy; ^7^Soft-Tissue, Peritoneum and Melanoma Surgical Oncology Unit, Istituto Oncologico Veneto - Istituto di Ricovero e Cura a Carattere Scientifico (IOV-IRCCS), Padua, Italy; ^8^Department of Statistical Sciences, University of Padova, Padua, Italy; ^9^Department of Surgery, Oncology and Gastroenterology, University of Padova, Padua, Italy

**Keywords:** melanoma, quality assurance, healthcare services and policy, survival analysis, real-word data analyses

## Abstract

**Introduction:**

Promoting standardization and quality assurance (QA) in oncology on the strength of real-world data is essential to ensure better patient outcomes. Wide excision after primary tumor biopsy is a fundamental step in the therapeutic pathway for cutaneous malignant melanoma (CMM). The aim of this population-based cohort study is to assess adherence to wide local excision in a cohort of patients diagnosed with CMM and the impact of this recommended procedure on overall and disease-specific survival.

**Materials and Methods:**

This retrospective cohort study concerns CMM patients diagnosed in the Veneto region (north-east Italy) in 2017, included in the high-resolution Veneto Cancer Registry, and followed up through linkage with the regional mortality registry up until February 29th, 2020. Using population-level real-world data, linking patient-level cancer registry data with administrative records of clinical procedures may shed light on the real-world treatment of CMM patients in accordance with current guidelines. After excluding TNM stage IV patients, a Cox regression analysis was performed to test whether the completion of a wide local excision was associated with a difference in melanoma-specific and overall survival, after adjusting for other covariates.

**Results:**

No wide excision after the initial biopsy was performed in 9.7% of cases in our cohort of 1,305 patients. After adjusting for other clinical prognostic characteristics, Cox regression revealed that failure to perform a wide local excision raised the hazard ratio of death in terms of overall survival (HR = 4.80, 95% CI: 2.05–11.22, *p* < 0.001) and melanoma-specific survival (HR = 2.84, 95% CI: 1.04–7.76, *p* = 0.042).

**Conclusion:**

By combining clinical and administrative data, this study on real-world clinical practice showed that almost one in ten CMM patients did not undergo wide local excision surgery. Monitoring how diagnostic-therapeutic protocols are actually implemented in the real world may contribute significantly to promoting quality improvements in the management of oncological patients.

## Introduction

The global incidence of cutaneous malignant melanoma (CMM) has steadily increased over the last few decades, resulting in new clinical and public health issues (1, 2). In this epidemiological setting, clinicians have been involved in updating their diagnostic and therapeutic procedures, while policymakers have been striving to balance the priority of ensuring access to the most effective therapies with the sustainability of healthcare systems.

The purpose of evidence-based CMM protocols is to provide high-quality guidance on patient management as well as scientific support for the most effective allocation of financial resources. In clinical practice, however, various situations may give rise to inconsistencies (even significant) between “canonical” guidelines and their real-world implementation. The recording and analysis of these multifaceted settings may provide important insight into how oncological protocols are actually applied in clinical practice, potentially helping to improve quality assurance (QA).

The surgical resection of CMM is the earliest step in a patient's treatment. After a histological assessment of primary cutaneous lesions, current diagnostic-therapeutic protocols (DTPs) recommend that excision margins (including surrounding skin and subcutaneous tissue) be wider the greater the melanoma's Breslow thickness. Excessively narrow excision margins increase the risk of both local recurrences and in-transit metastases (3, 4), while excessively wide resections may result in greater morbidity and higher healthcare costs (5). It has also been demonstrated that high-quality surgical procedures improve patients' short-term survival and lower the costs of their care (6, 7).

Although surgical excision has long been recognized as the treatment of choice for improving the chances of survival for CMM patients, the real burden of failing to carry out this procedure remains unknown, and specific real-world data on the impact on survival is limited. Using population-level real-world data, linking patient-level cancer registry data with administrative records of clinical procedures may shed light on the real-world treatment of CMM patients in accordance with current DTPs.

In a consecutive, population-based series of primary CMMs, this retrospective cohort study examines the level of adherence to guideline-advised wide local excision after diagnosis and the impact of this procedure on short-term overall and melanoma-specific survival.

## Methods

### Context

The Italian National Health System is a public system mainly financed by general taxation, and essentially organized on a regional basis (8). Its policies are grounded in the fundamental values of universality, free access, freedom of choice, pluralism in provision, and equity.

The Regional Authority for the Veneto (in north-east Italy) has adapted national guidelines on melanoma to the local context and established a diagnostic and therapeutic patient care pathway that aims to achieve the best patient outcomes while reducing inequalities and unwarranted variability in patient management and ensuring the healthcare system's sustainability (9).

In 2017, the Veneto Tumor Registry set up a high-resolution registry of CMM cases in collaboration with the Veneto Oncology Network. Based on patients' clinical records, this high-resolution cancer registry retrospectively collects data on the clinical features and stage of tumors at diagnosis (10).

### Data and Variables

This retrospective cohort study considered patients living in Veneto who were diagnosed with CMM in 2017, as recorded in the high-resolution Veneto Cancer Registry in that year. Stage IV patients were excluded, since enlargement surgery after primary excision is only indicated in patients with a single visceral metastatic lesion or a limited number of metastases mainly involving the soft tissues, and we had no data at this level. This was the only exclusion criterion adopted in the study.

Among the variables recorded in the high-resolution registry, the clinical and pathological characteristics of the melanoma lesions at diagnosis potentially associated with survival that were considered in the present study were, in particular: (a) demographics (age and gender); (b) tumor-infiltrating lymphocytes ([TILs] absent vs. present); (c) number of mitoses (≤2 vs. >2/mm^2^); (d) growth phase (radial vs. vertical); (e) Breslow thickness ( ≤0.75, 0.76–1.50, 1.51–3.99, ≥4.00); (f) combined clinical and pathological TNM stage at diagnosis (I, II, III, IV); (g) ulceration (absent vs. present); (h) regression (absent vs. present) and histological subtype (malignant melanoma, superficial spreading melanoma, nodular melanoma, lentigo maligna, acral-lentiginous melanoma, desmoplastic melanoma, and spitzoid melanoma).

The Veneto Cancer Registry, which is linked to regional administrative databases, records all diagnostic and therapeutic procedures (surgical and non-surgical) listed in hospital discharge records, including outpatient procedures. The time of patients' enrollment in this study always coincided with the diagnostic excision of their primary melanoma.

The vital status of all subjects was determined by linking their records to the regional Mortality Registry (available up to February 29th, 2020). The population file of residents, as made available by the regional Healthcare System up to December 31st, 2020, was used to identify patients who were lost at follow-up because they moved away from Veneto. In the archives used for this study (resident population records, regional cancer registry, regional mortality registry), individuals are always identified by a unique number. This number was used for record linkage purposes, so we are confident that the efficacy of the record linkage process was satisfactory.

### Assessing Real-World Data to Improve Patient Awareness

The study's findings were discussed by an interdisciplinary board of epidemiologists, healthcare administrators, pathologists, and surgeons.

### Statistical Analyses

Frequencies and percentages were used for the descriptive analysis. The association between categorical variables was tested with the chi-squared test or Fisher's exact test, as appropriate. Cumulative survival rates were calculated with the Kaplan-Meier method by stage, taking the wide local excision group as a reference. The log-rank test was used to test the difference in survival rates between the wide local excision groups. All cases were entered into the study at the date of incidence and were followed-up until February 29th, 2020, or the date of death or exit from the cohort due to relocation, whichever happened first. A Cox regression analysis was run to test whether wide local excision was associated *per se* with overall and melanoma-specific survival after adjusting for covariates: gender, age at diagnosis, tumor stage at diagnosis, TILs, growth phase, mitoses, ulceration, regression, and histological subtype. The Cox model for melanoma-specific survival was not adjusted for growth phase because this variable perfectly predicts the outcome (no melanoma-related deaths occurred among patients with a radial growth pattern). The Breslow thickness variable was not included in the Cox model to avoid an over-adjustment, because it is already taken into account in the stage variable. In the Cox regression analysis, we grouped the less frequent histology categories (acral-lentiginous, lentigo maligna, desmoplastic, and spitzoid) in the “Other” modality. The hazard ratio was given with its 95% confidence interval. The missing data were not inferred, and only patients with full data sets were entered in the Cox regression model. The adequacy of the proportionality assumption in the Cox regression was assessed based on weighted residuals.

The R 3.5.2 statistical package was used to link records and for all statistical analyses. The significance level was set at 5%.

### Ethics

The data analysis was carried out on anonymized aggregate data, with no possibility of identifying individuals. Ethical approval for the study was obtained from the Veneto Oncological Institute's Ethics Committee (No. 52/2016).

## Results

After excluding stage IV patients at diagnosis (*n* = 63), 1,305 Veneto residents were diagnosed with CMM in 2017. They were evenly distributed by gender (males 52.95%; females 47.05%), with a median age of 60 years (48–73). In this cohort, 126 patients (9.66%) were not treated further with wide excision after their initial biopsy.

Table 1 details the clinical characteristics of all patients, differentiating between those who underwent wide excision and those who did not. There was a significant association between age and the likelihood of undergoing additional surgery (*p* < 0.001), with older people, and especially those over 80, being less likely to do so. In particular, 41.3% of patients over the age of 80 did not have further surgery, compared with 7.9% of those in the 50–59 age group, 9.5% in the 40–49 age group, and 5.6% of those under 40. A lower proportion of patients undergoing additional surgery were found to also have the following histopathological features: a radial growth phase, a greater Breslow thickness (especially over 4.0 mm), the absence of TILs, a mitotic rate >2.0 mm^2^, the presence of ulceration, nodular melanoma, and TNM stage II and III at diagnosis.

**Table 1:** Demographics and pathology of patients with cutaneous malignant melanoma (CMM) who did or did not undergo wide local excision (TILs, tumor infiltrating lymphocytes; TNM, CMM staging).

		**Wide excision**		**Chi-squared test**
	**Total**	**Yes**	**No**	***p-value***
	***N* (% column)**	***N* (% column)**	***N* (% column)**	
**All patients**	1,305 (100)	1,179 (90.34)	126 (9.66)	–
**Gender**				0.601
Male	691 (52.95)	621 (52.67)	70 (55.56)	
Female	614 (47.05)	558 (47.33)	56 (44.44)	
**Age (years)**				<0.001
<40	132 (10.11)	125 (10.60)	7 (5.56)	
40–49	243 (18.62)	231 (19.59)	12 (9.52)	
50–59	253 (19.39)	243 (20.61)	10 (7.94)	
60–69	237 (18.16)	220 (18.66)	17 (13.49)	
70–79	270 (20.69)	242 (20.53)	28 (22.22)	
80+	170 (13.03)	118 (10.01)	52 (41.27)	
**Growth phase**				0.040
Radial	270 (20.69)	253 (21.46)	17 (13.49)	
Vertical	786 (60.23)	709 (60.14)	77 (61.11)	
Missing	249 (19.08)	217 (18.40)	32 (25.40)	
**Breslow thickness**				<0.001
≤ 0.75	654 (50.12)	611 (51.82)	43 (34.13)	
0.76–1.50	275 (21.07)	259 (21.97)	16 (12.70)	
1.51–3.99	199 (15.25)	182 (15.44)	17 (13.49)	
≥4.00	132 (10.11)	90 (7.63)	42 (33.33)	
Missing	45 (3.45)	37 (3.14)	8 (6.35)	
**TILs**				0.006
Absent	309 (23.68)	271 (22.98)	38 (30.16)	
Present	846 (64.83)	780 (66.16)	66 (52.38)	
Missing	150 (11.49)	128 (10.86)	22 (17.46)	
**Mitotic rate (mm** ^**2**^ **)**				<0.001
0–2	857 (65.67)	804 (68.19)	53 (42.06)	
>2	328 (25.13)	278 (23.58)	50 (39.68)	
Missing	120 (9.20)	97 (8.23)	23 (18.26)	
**Ulceration**				<0.001
Absent	1,016 (77.85)	949 (80.49)	67 (53.17)	
Present	237 (18.16)	185 (15.69)	52 (41.27)	
Missing	52 (3.99)	45 (3.82)	7 (5.56)	
**Regression**				0.001
Absent	615 (47.13)	553 (46.90)	62 (49.21)	
Present	409 (31.34)	385 (32.66)	24 (19.04)	
Missing	281 (21.53)	241 (20.44)	40 (31.75)	
**Histological subtype**				<0.001^*^
Malignant melanoma	82 (6.28)	74 (6.28)	8 (6.35)	
Superficial spreading melanoma	943 (72.26)	877 (74.39)	66 (52.38)	
Nodular melanoma	190 (14.56)	151 (12.81)	39 (30.95)	
Lentigo maligna	32 (2.45)	27 (2.29)	5 (3.97)	
Acral-lentiginous melanoma	22 (1.69)	17 (1.44)	5 (3.97)	
Desmoplastic melanoma	6 (0.46)	5 (0.42)	1 (0.79)	
Spitzoid melanoma	30 (2.30)	28 (2.37)	2 (1.59)	
**TNM (at enrollment)**				<0.001
I	854 (65.44)	802 (68.03)	52 (41.27)	
II	215 (16.48)	175 (14.84)	40 (31.75)	
III	141 (10.80)	121 (10.26)	20 (15.87)	
Missing	95 (7.28)	81 (6.87)	14 (11.11)	

* *Fisher's exact test*.

**Figure 1:**
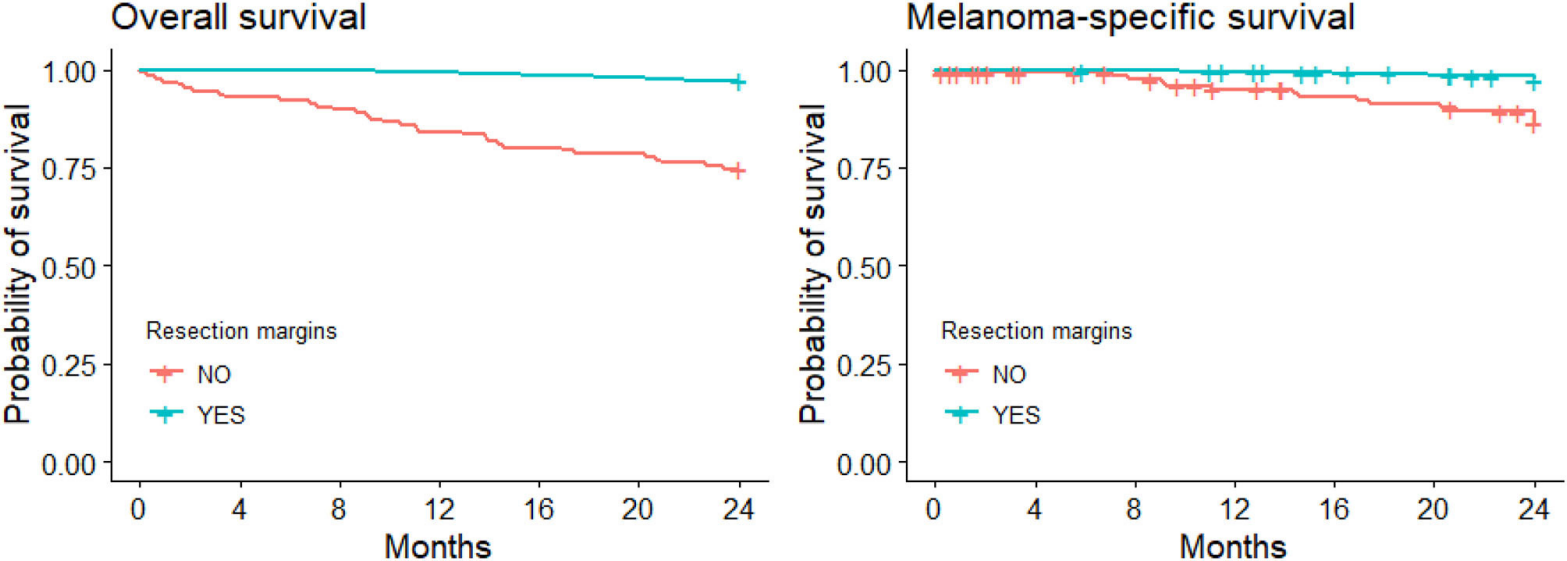
Kaplan-Meier curves for overall and melanoma-specific survival with/without wide local excision.

**Figure 2:**
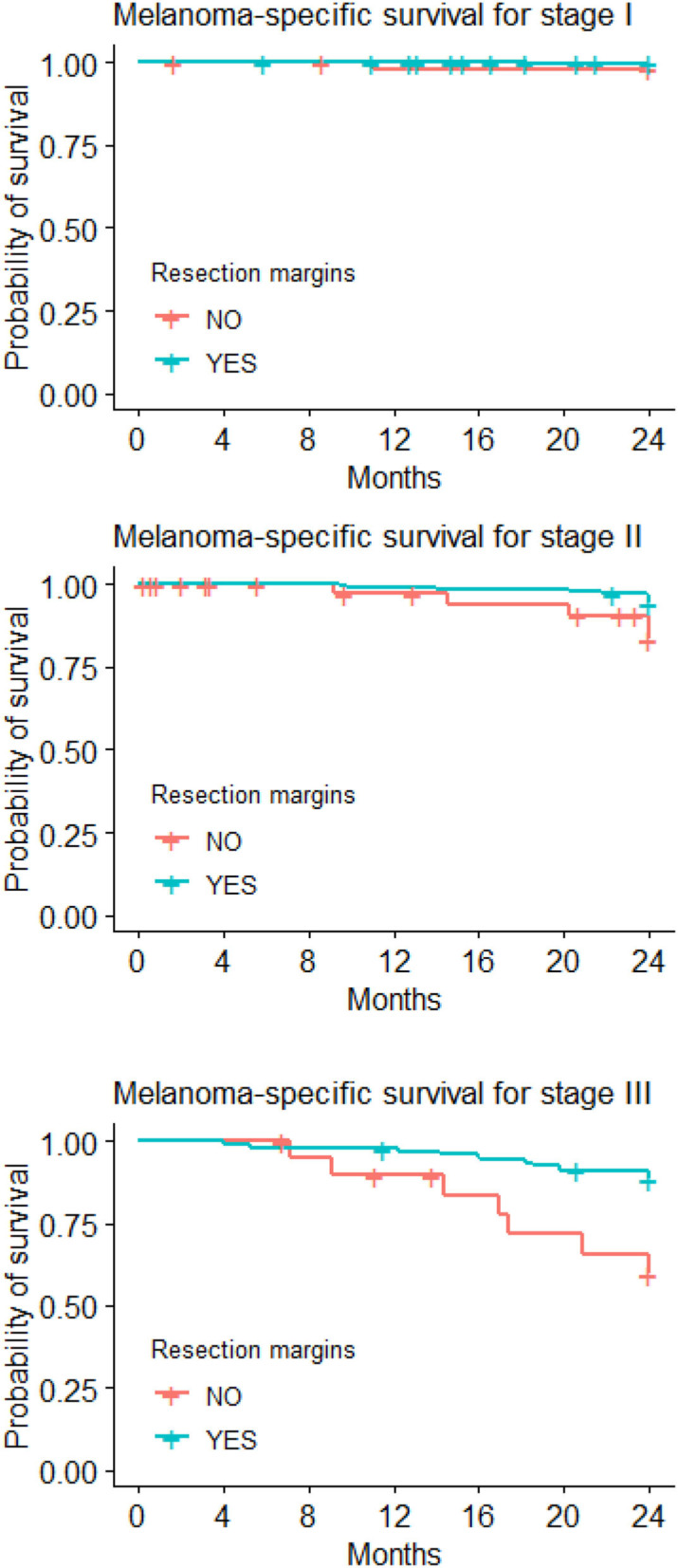
Kaplan-Meier curves for melanoma-specific survival with/without wide local excision.

Sixty-six CMM patients died during the follow-up (44 of them due to melanoma). Figure 1 illustrates the Kaplan-Meier comparison of overall (OS) and melanoma-specific survival (MSS) by surgical management of the primary lesion. Figure 2 depicts the Kaplan-Meier curves for melanoma-specific survival by stage and treatment with or without wide excision; the differences were significant for stage II and stage III (*p* = 0.030 and *p* = 0.001).

Table 2 shows the Kaplan-Meier survival estimates at 2 years after diagnosis, by wide local excision group and patients' demographic, clinical, and anatomopathological characteristics.

**Table 2:** Bivariate analyses of 2-year survival (Kaplan Meier analysis) by clinical-histological characteristics and wide excision group.

	**Total survival (95% CI)**	**Wide excision**	
		**Yes**	**No**
		**Survival (95% CI)**	**Survival (95% CI)**
All patients	94.9 (93.6–96.1)	97.1 (96.0–98.0)	74.6 (66.1–81.9)
**Gender**
Male	94.2 (92.2–95.8)	96.1 (94.3–97.5)	77.1 (65.6–86.3)
Female	95.8 (93.9–97.2)	98.2 (96.7–99.1)	71.4 (57.8–82.7)
**Age (years)**
<40	100.0 (97.2–100.0)	100.0 (97.1–100.0)	100.0 (59.0–100.0)
40–49	99.6 (97.7–99.9)	100.0 (98.4–100.0)	91.7 (61.5–99.8)
50–59	99.2 (97.2–99.9)	99.2 (97.1–99.9)	100.0 (69.2–100.0)
60–69	97.5 (94.6–99.0)	97.7 (94.8–99.3)	94.1 (71.3–99.9)
70–79	91.8 (87.9–94.8)	93.0 (89.0–95.9)	82.1 (63.1–93.9)
80+	79.4 (72.5–85.2)	91.5 (85.0–96.0)	51.9 (37.6–66.0)
**Growth phase**
Radial	98.9 (96.8–99.8)	98.8 (96.6–99.8)	100.0 (80.5–100.0)
Vertical	94.9 (93.1–96.3)	97.2 (95.7–98.3)	74.1 (62.8–83.4)
**Breslow thickness**
≤ 0.75	98.5 (97.2–99.3)	98.7 (97.4–99.4)	95.3 (84.2–99.4)
0.76–1.50	97.5 (94.8–99.0)	98.1 (95.6–99.4)	87.5 (61.7–98.4)
1.51–3.99	93.9 (89.7–96.8)	95.6 (91.5–98.0)	76.5 (50.1–93.2)
≥4.00	76.5 (68.4–83.5)	88.9 (80.5–94.5)	50.0 (34.2–65.8)
**TILs**
Absent	93.2 (89.8–95.7)	95.9 (92.9–98.0)	73.7 (56.9–86.6)
Present	96.6 (95.1–97.7)	97.8 (96.5–98.7)	81.8 (70.4–90.2)
**Mitotic rate (mm2)**
0–2	97.9 (96.7–98.8)	98.4 (97.2–99.1)	90.6 (79.3–96.9)
>2	88.7 (84.8–91.9)	93.9 (90.4–96.4)	60.0 (45.2–73.6)
**Ulceration**
Absent	97.8 (96.7–98.6)	98.3 (97.3–99.0)	91.0 (81.5–96.7)
Present	83.5 (78.2–88.0)	91.9 (87.0–95.4)	53.8 (39.5–67.8)
**Regression**
Absent	94.6 (92.5–96.3)	97.6 (96.0–98.7)	67.7 (54.7–79.0)
Present	97.1 (94.9–98.5)	98.2 (96.3–99.3)	79.2 (57.8–92.9)
**TNM (at enrollment)**
I	98.2 (97.1–99.0)	98.5 (97.4–99.2)	94.2 (84.1–98.8)
II	89.8 (84.9–93.5)	96.0 (91.9–98.4)	62.5 (49.2–79.5)
III	84.4 (77.3–90.0)	89.3 (82.3–94.2)	55.0 (31.5–76.9)
**Histological subtype**
Malignant melanoma	91.5 (83.2–96.5)	94.6 (86.7–98.5)	62.5 (45.8–77.3)
Superficial spreading melanoma	96.6 (95.2–97.7)	97.7 (96.5–98.6)	81.8 (70.4–90.2)
Nodular melanoma	87.8 (82.4–92.2)	94.7 (89.8–97.7)	61.5 (46.6–76.6)
Lentigo maligna	96.9 (83.8–99.9)	96.3 (81.2–99.9)	100.0 (47.8–100.0)
Acral-lentiginous melanoma	90.9 (70.8–98.9)	100.0 (80.5–100.0)	60.0 (14.7–94.7)
Desmoplastic melanoma	83.3 (35.9–99.6)	80.0 (28.4–99.5)	100.0 (2.5–100.0)
Spitzoid melanoma	100.0 (88.4–100.0)	100.0 (87.7–100.0)	100.0 (15.9–100.0)

With regard to the Cox regression (Table 3) only patients with full data sets were entered in model (70% of the cohort). The patients excluded from the analysis experienced a survival probability (88.2%) instead those included in the multivariate analysis (91.3%). After adjusting for other clinical prognostic factors, failure to perform a wide local excision increased the hazard ratio of death in terms of overall survival (HR = 4.80, 95% CI: 2.05–11.22, *p* < 0.001) and melanoma-specific survival (HR = 2.84, 95% CI: 1.04–7.79, *p* = 0.042). The proportionality assumption was accepted for both overall (*p* = 0.52) and melanoma-specific survival (*p* = 0.48). Cox models were also used to evaluate the association between a lack of wide excision and overall survival, stratified by stage, and adjusted for gender, and age. The hazard ratios were 2.50 for stage I (95% CI: 0.65–9.62, *p* = 0.184), 4.20 for stage II (95% CI: 1.63–10.82, *p* = 0.003), and 2.92 for stage III (95% CI: 1.10–7.78, *p* = 0.032). The proportionality assumption was accepted for all models (*p* = 0.37 for stage I, *p* = 0.75 for stage II, and *p* = 0.24 for stage III) [data not shown]. A sensitivity analysis, including only variables without missing data (age, sex, and histological subtype), revealed an hazard ratio of death in terms of overall survival (HR= 2.59, 95% CI: 1.66–4.03, *p* = < 0.001) and melanoma-specific survival (HR = 22.22, 95% CI: 1.14–4.33, *p* = 0.0196) [data not shown].

**Table 3:** Cox regression model for overall and melanoma-specific survival (HR, hazard ratio).

		**Overall survival^°^**			**Melanoma-specific survival^°°^**		
		**HR**	**95% CI**	***P***	**HR**	**95% CI**	***P***
**Wide excision** (reference: yes)	No	4.80	2.05–11.22	<0.001	2.84	1.04–7.76	0.042

° *Adjusted for gender, age, stage of disease at diagnosis, mitotic rate, growth phase, TILs, ulceration, regression, and histological subtype*.

°° *Adjusted for gender, age, stage of disease at diagnosis, mitotic rate, TILs, ulceration, regression, and histological subtype*.

## Discussion

The need for a wide(r) excision of CMMs following an initial biopsy is well established, and it is consistently recommended that this surgical procedure attempts to not only remove any residual primary tumor, but also, if possible, to identify any proximal micro-metastatic disease (11–13). This population-based study investigated adherence to guideline-advised wide local excision following a diagnosis of CMM, and the impact of this procedure on overall and disease-specific survival in real-world oncology practice. Roughly one in every ten of the 1,305 CMM patients in our cohort did not have the recommended surgical procedure, which had a negative effect on their short-term overall and melanoma-specific survival. To the best of our knowledge, no modern randomized-control trial or observational study has been conducted to assess the impact of failing to perform this procedure. A recent open-label multicenter trial compared overall and melanoma-specific survival after narrow (1 cm) and wide (3 cm) excision margins had been obtained. At a median follow-up of 8.8 years, the study found the risk of death from melanoma or any other cause higher in the narrow-margin group than in the wide-margin group, though the difference for any cause mortality was not significant (14). While no comparison was drawn directly with no excision, the fact that wide local excision margins were associated with better survival chances than narrow excision margins suggests that the difference would have been even more in favor of wide local excision as opposed to no surgical procedure.

Both patients and physicians have to be taken into consideration when addressing performance in the multifaceted setting of real-world clinical practice (15). In the case of CMM, physicians (whether dermatologists, general practitioners, or surgeons) may fail to inform their patients about the vital importance of a “wider resection.” A patient-centered approach is required for effective healthcare, which basically entails collaborative patient-physician relationships, clear communication, and choosing the right time to provide information (16). Further studies should focus on how to improve healthcare professionals' communication skills in an oncological setting.

This research does not address why some patients did not undergo a wide excision procedure for their melanoma. Even well-informed patients may have their reasons for refusing to follow their doctors' recommendations, while there may also be other factors at play that we were unable to investigate thoroughly. In the present sample, older people, particularly those over the age of 80, were associated with a lower likelihood of undergoing additional surgery. This could be due to differences in their perceptions of health priorities, difficulties in accessing the necessary healthcare services, financial concerns, or cultural and psychological attitudes (17). On the other hand, gender did not seem to influence the likelihood of undergoing additional surgery. Some histopathological features were also linked to a higher likelihood of no additional surgery. Promoting patient compliance with best clinical practice should include empowering patients and emphasizing the fundamental importance of their choices. In keeping with this approach, Wagner et al. emphasized that a successful disease management program should include an elective focus on the efficacy of communication between caregivers and patients (18). For instance, a plain sentence in the pathology report should read: “For your safety, please communicate this report to your dermatologist or your general practitioner.”

Monitoring the performance of healthcare services by linking all healthcare databases is critical to improving cancer patients' healthcare management and outcomes (19).The increasing importance of quality assurance (QA) in the clinical management of CMM is underpinned by the steady growth of national and regional melanoma registries [such as the Danish Metastatic Melanoma Database (20) and the Dutch Melanoma Treatment Registry]. Among other benefits, the medical, surgical, and pathological information (21) that they record can promote transparency in melanoma care, shed light on the real-world cost-effectiveness of clinical procedures, support policymakers' decisions, and make useful information available for clinical trials. The use of real-world registry-based data in this study can serve as an example of how monitoring adherence to diagnostic-therapeutic procedures can help promote quality assurance (QA) and, ultimately, improve patient outcomes.

This study's main strength is that it is based on real-world data collected at the population level by a regional healthcare system. Data on real clinical cases can provide crucial information on how clinical procedures perform outside of the confines of research settings. The study also has some limitations, however. In particular, the biological profiling of CMMs could be expanded further, and the costs of each of the major clinical interventions quantified. Moreover, this research does not provide conclusive answers as to why some patients did not undergone wide excision following primary tumor biopsy. The groups identified from this study as having a lower likelihood of wide excision after diagnosis—particularly the elderly—could be the focus of future research, possibly using qualitative methods to investigate the reasons for these disparities. Finally, it is worth noting that some information is missing from our database regarding histopathological variables for some patients. While we are not aware of any particular bias relating to the completeness of our data, and our sensitivity analysis indicated a consistency in the results regarding melanoma-specific survival, the overall survival hazard ratio obtained with the sensitivity analysis amounted to about half the ratio obtained with the fully-adjusted model. This latter finding could be due to the sensitivity analysis failing to adjust for all potential confounders or to a selection bias in the fully adjusted model because patients with missing data were included, or even to a combination of the two.

In conclusion, this study demonstrates the importance of monitoring the quality of CMM surgery and shows that omitting wide local excision results in worse short-term survival. These findings also highlight the crucial importance of patient empowerment in the clinical management of their own oncological disease.

## Data Availability Statement

The dataset generated during and/or analysed during the current study are available from the corresponding author on reasonable request.

## Ethics Statement

The studies involving human participants were reviewed and approved by Istituto Oncologico Veneto Ethical Commitee. To ensure confidentiality and anonymity, the Veneto Regional Authority removes all direct identifiers and replaces them with a code number in all datasets to retain the opportunity to link data from different administrative databases. In this case according to No. 9/2016 of the Italian Guarantor for the Protection of Personal Data it is possible do not collect written consent from patients.

## Author Contributions

AB and GDa conceived the presented idea. MZ, RS, AV, PD, and SM collected the data, verified its accuracy, and developed the methods. CD verified the analyses. AB and RF wrote the draft. MR, GDe, and AB supervised the project and revised the draft. CR found the financial support for the project. All authors read and approved the final manuscript.

## Conflict of Interest

This study was funded by CARIPARO, Fondazione Cassa di Risparmio di Padova e Rovigo. The foundation had no role in the study's design, the collection, analysis, or interpretation of the data, the writing of the manuscript, or the decision to submit the paper for publication. The authors declare that the research was conducted in the absence of any commercial or financial relationships that could be construed as a potential conflict of interest.

## Publisher's Note

All claims expressed in this article are solely those of the authors and do not necessarily represent those of their affiliated organizations, or those of the publisher, the editors and the reviewers. Any product that may be evaluated in this article, or claim that may be made by its manufacturer, is not guaranteed or endorsed by the publisher.
